# New outcomes on how silicon enables the cultivation of *Panicum maximum* in soil with water restriction

**DOI:** 10.1038/s41598-022-05927-z

**Published:** 2022-02-03

**Authors:** Juan Ricardo Rocha, Renato de Mello Prado, Marisa de Cássia Piccolo

**Affiliations:** 1 Laboratory of Plant Nutrition, Department of Agricultural Production Sciences-Soil and Fertilizer Sector, School of Agricultural and Veterinarian Sciences, São Paulo State University (UNESP), Prof. Paulo Donato Castellane Avenue, Jaboticabal, SP 14884900 Brazil; 2 Laboratory of Nutrient Cycling, Center of Nuclear Energy in Agriculture, University of São Paulo (USP), 303 Centenário Avenue, Piracicaba, SP 13400970 Brazil

**Keywords:** Climate-change ecology, Ecophysiology, Grassland ecology

## Abstract

Climate change increases the occurrence of droughts, decreasing the production of tropical forages through the induction of physiological stress. Si is expected to broaden the limit from physiological stress of forages grown under water restriction, which may come from an improvement in the stoichiometric homeostasis of Si with N and C, favoring physiological aspects. This study assessed whether Si supply via fertigation improves physiological aspects and the water content in the plant by means of an antioxidant defense system and changes in the C:N:Si stoichiometry during the regrowth of two cultivars of *Panicum maximum* grown under two soil water regimes (70 and 40% of the soil’s water retention capacity). The forages studied are sensitive to water deficit without silicon supply. The application of Si via fertigation attenuated the water deficit, favoring plant growth by stabilizing the stoichiometric homeostasis C:N and C:Si, which are responsible for increasing the plant capacity of converting accumulated C in dry mass, favoring the water content of the plant tissue and the photosynthetic efficiency. This study highlights the importance of the physiological function of Si, and effects on the stoichiometry of C and N, which are neglected in most research on forages grown under water restriction.

## Introduction

Pasture (Poacea) is the main food source for livestock in the tropical regions of the world^1,2^ due to its high biomass production capacity and acceptability to animals^3^, but it is sensitive to drought^4^. Abiotic stresses such as drought^5^ and the high temperature^6^ threatens its biomass production capacity^7^. Water is, therefore, the main limiting factor for agricultural production^8^, and the impacts of climate change can aggravate stress^9,10^ promoting physiological disturbances in photosynthesis, loss of integrity of the membrane and generation of reactive oxygen species (ROS)^11,12^. It is noteworthy that stoichiometric homeostasis is useful to explain and predict the responses of different plants to stresses such as drought^13^.

Under drought, plants activate their defense system to minimize damage. This is even more evident in species that absorb silicon, that is, they have a high capacity to capture and transport Si to the leaves^14,15^. Most studies so far on Si and water deficit in different species have discussed the effect of this element in reducing the transpiration rate, which increases the efficiency of water use^16–18^, the regulation of multiple defenses antioxidants^19^, pigment levels^20^ and photosynthetic parameters^21,22^. Studies have suggested that Si reduces water stress due to less water loss by cuticular transpiration^23^, but the cuticular transpiration rate is very low compared to stomatal transpiration^24^, suggesting that Si also affects other mechanisms that need to be better investigated. Recent research has indicated that Si absorption improves its physiological aspects mainly by modifying elemental stoichiometry and could minimize damage from water deficit^25–27^. However, most studies on stoichiometric homeostasis focus mainly on N and P, studies on the Si are incipient^28,29^.

These aspects are important because plants under water deficit have energy requirements for their metabolism, with a limitation in CO_2_ assimilation rate accompanied by an increase in the activity of another sink of absorbed energy, for example, photorespiration^30^. In this scenario, nutrient uptake is also affected, impairing plant metabolism and disturbing the stoichiometric homeostasis of C, N, and Si in plant tissues under water deficit^31^. However, the use of Si can favor elementary stoichiometric homeostasis by interfering with the composition of the cell, especially the cell wall. Noteworthy, most of the Si absorbed (90%) by the plant is in the form of amorphous silica, which is mostly used in Si-cellulose structures in the cell wall^32^. Complexation of Si with cell wall macromolecules is likely to occur by sugar stabilization, in a similar way to that of borate-mediated formose reaction^33^. Silicon thus binds, for example, to hemicellulose components via Si–OC bonds, forming an organosilicate compound^34^.

Despite the lack of studies on Si, this nutrient requires less energy to be incorporated into leaf tissues than C. It can thus replace part of the C in cell wall organic compounds such as cellulose and hemicellulose, mainly due to its high permeability in lipid bilayers^35^. The effect of Si in improving C assimilation by plants is because a large part of it can be incorporated into photosynthetically active tissue cells at the expense of stabilizing compounds^36^. These energy benefits of Si favor plant metabolism, that is, the stoichiometric homeostasis of C and N. This in turn may increase C use efficiency and consequently C conversion into biomass, i.e., plant growth under water deficit.

According to literature reports, Si absorption varies depending on the genetic factor^37^; However, further investigations should clarify whether this benefit occurs in other pasture species such as *Panicum maximum*. This specie is widely cultivated worldwide, especially due to its regrowth capacity. Studies show that silicon reduces reducing oxidative stress by increasing the synthesis of antioxidant compounds^38,39^, but these benefits may be due to the improvement of elementary stoichiometric (C:N) homeostasis. Therefore, further research is needed to prove this assumption.

In this context, the hypothesis should be tested whether Si can attenuate water deficit because plants can tolerate a higher level of physiological stress after stoichiometric homeostasis has been improved. This improvement would change the C:N and C:Si ratios, consequently affecting the capacity of plants to convert cumulative C into dry matter. These elements in turn would favor some physiological processes and the growth of two cultivars of *P. maximum*.

This information might help in the systematic assessment of the impacts of Si accumulation on the increase of the limit of physiological stress of plants due to its effect on the stoichiometry of C and N in pastures. Also, it provides may serve as a reference for the management of pastures under water restriction.

To test the hypothesis, this study assess whether Si supply via fertigation improves physiological aspects and water content in the plant by involving the antioxidant defense system. In addition, the study analyzes whether Si supply modifies the C:N:Si stoichiometry and the regrowth performance of two *P. maximum* cultivars grown under two soil water regimes (70 and 40% of soil water holding capacity).

## Material and methods

### Plant material and growth conditions

Two trials were carried out at São Paulo State University (UNESP), in Jaboticabal, Brazil in the year 2020, concerning the second growth cycle or the regrowth of *P. maximum* cultivars Massai (experiment 1) and BRS Zuri (experiment 2).

Seeds of *P. maximum*, were obtained from the Brazilian Agricultural Research Corporation of the Ministry of Agriculture, Livestock and Food Supply, Brazil (registered and protected by the Ministry of Agriculture, Livestock and Supply—MAPA). This research was not conducted with endangered species and was the is accordance with the Declaration of IUCN Policy on Research Involving Endangered Species.

**Figure 1:**
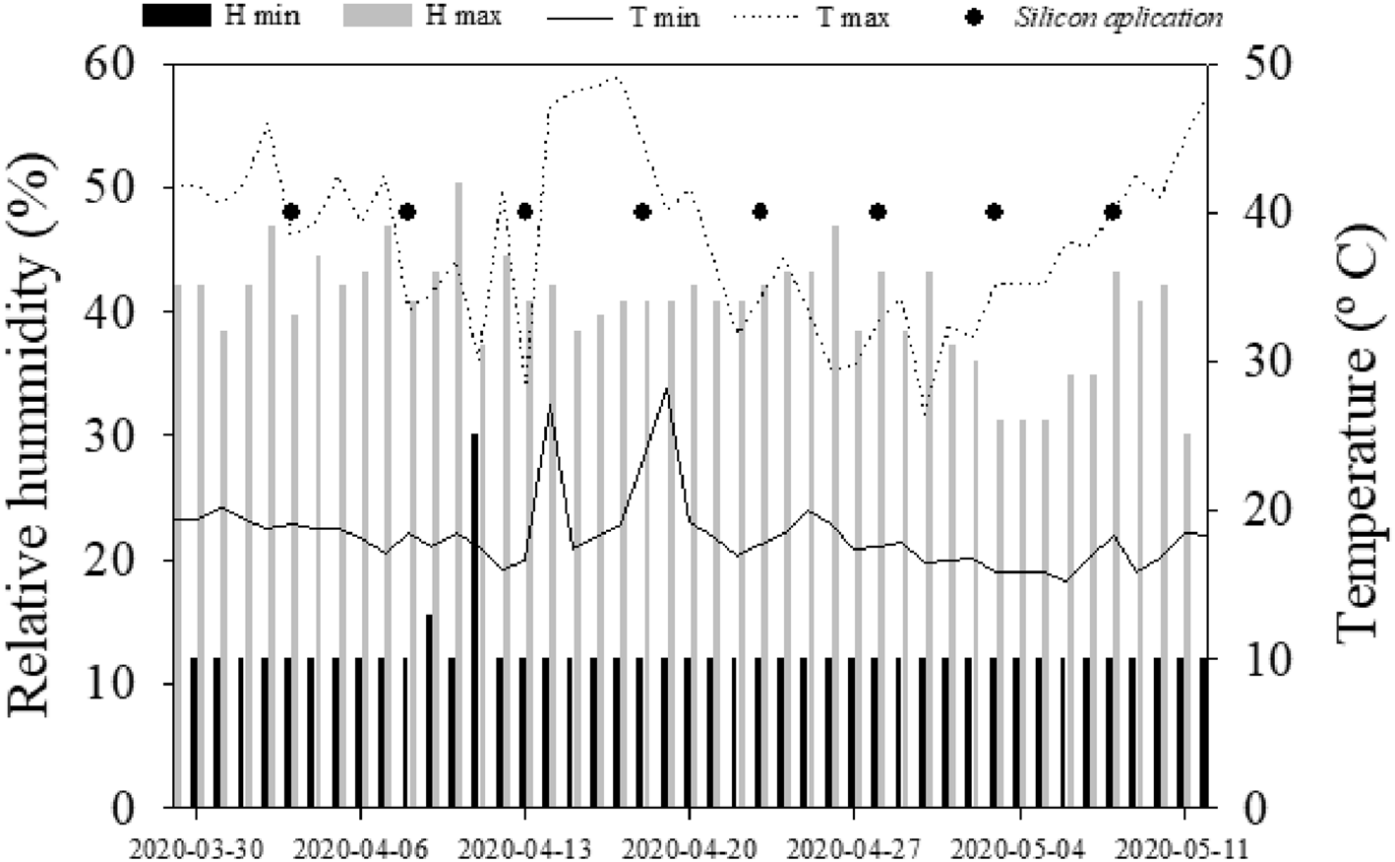
Maximum and minimum air temperature and humidity of the greenhouse and silicon application during the experimental period. *H min* minimum humidity, *H max* maximum humidity, *T min* minimum temperature, *T max* maximum temperature.

During the development of the study, meteorological data were collected daily, namely temperature (T) and relative air humidity (H) in the place of cultivation of the experiments, using a thermohygrometer (Fig. 1).

### Experimental design and treatments

In both experiments, the treatments were arranged in a 2 × 2 factorial scheme, with the application of Si via fertigation (root) and the control (without application of Si) combined with two water regimes at 70% and 40% of soil water retention capacity, arranged in randomized blocks with six repetitions. The experimental unit consisted of a 7 dm^3^ vessel filled with 6 dm^3^ of samples from an Entisol (Quartzipsamment).

Chemical analysis of the soil was carried out for fertility purposes according to the method described by Raij et al.^40^. The following results were found: pH CaCl_2_ = 4.3; O.M. (organic matter) = 9 g dm^−3^; P in resin extractor = 2 mg dm^−3^; S = 18 mg dm^−3^; Ca = 3 mmol dm^−3^; Mg = 1 mmol dm^−3^; K = 0.3 mmol dm^−3^; Al = 0 mmol dm^−3^; H + Al = 16 mmol dm^−3^; SB (sum of bases) = 4; CEC (cation exchange capacity) = 20 mmol_c_ dm^−3^; V (base saturation) = 21%. The available Si content was determined (3.0 mg dm^−3^) using the method described by Korndörfer^41^. Soil particle size distribution was determined using the method described by Gee and Or^42^, and the following results were found: 540 g kg^−1^ of sand, 380 g kg^−1^ of clay and 90 g kg^−1^ of silt.

Thirty days later, lime was applied to the soil to correct acidity, and to increase base saturation (K + Ca + Mg/K + Ca + Mg + H + Al) to 60%. Fertilization was carried out by applying 150 mg dm^−3^ of N, P and K, in the form of ammonium sulfate, triple superphosphate and potassium chloride, respectively, and 5 mg dm^−3^ of Zn in the form of zinc sulfate; they were all mixed to the volume of the soil.

The forage was first cut at 12 cm from the ground level at 45 days after uniform cut of the shoots. At 10 days after this cut, the second growth cycle of the forage started and the treatments were applied, using Si via fertigation and adapting the two study water availability regimes.

The source of Si was sodium silicate and potassium stabilized with sorbitol (113.4 g L^−1^ de Si and 18.9 g L^−1^ of K_2_O), in the concentration of 2.5 mmol L^−1^, as indicated by Birchall^43^, simulating a 5 mm irrigation depth every five days. The study of Rocha et al.^25^ on *Brachiaria* was used as a reference; Si was applied immediately after the first cycle of forage for a period of 40 days.

The levels of soil water availability were determined using the microporosity values found by the tension table method with a 60 cm high water column; this measurement was performed while considering soil density, which was determined by the ratio of soil dry weight in the greenhouse at 110 °C, for 24 h, and the volume of undisturbed soil sample^44^. Total microporosity was considered as equivalent to 100% of soil water retention capacity; however, the water condition was considered as 70% of this value, as it corresponds to the usual demand for most crops. The water deficit condition was achieved by maintaining the water level at 40% of the soil water retention capacity, determined on study of Rocha et al.^25^. Previous studies suggest that the best benefit of Si occurs in plants with a water level close to 40% and at a water level close to 70% it is sufficient to achieve maximum forage dry mass production. Water availability was controlled daily by the method of weighing the vessels after replacement of evapotranspiration water.

### Performed analyses

#### Phenolic compounds

Biological evaluations were carried out at 45 days after the application of the treatments. Leaf + 1 (first fully developed leaf) was collected from a tiller chosen at random to determine the content of total phenolic compounds, following the method described by Singleton and Rossi^45^.

#### Quantum efficiency of PSII (Fv/Fm)

To ensure the adaptation of the leaves to light, quantum yield of PSII was measured between 7 and 9 a.m., on the first fully developed leaf of each plant. Also, maximum variable fluorescence (Fv/Fm), which would be the maximum quantum efficiency of PSII, was determined using a portable fluorometer (Opti-sciences—Os30P)^46^.

#### Quantification of chlorophyll

Total chlorophyll index was determined using an indirect electronic chlorophyll meter (Clorofilog—Falker^®^ brand). Reading was performed in the middle third of the leaf blade of the first fully developed leaf, which uses three light frequency ranges; thus, the optical measurement analyzes the absorption of light by the leaf by estimating the presence of chlorophyll.

#### Electrolyte leakage index

Damage to cell membrane integrity was assessed using the method of determining the electrolyte leakage index (EL), proposed by Dionisio-Sese and Tobita^47^. Ten leaf discs (129 mm^2^) were collected from the first fully developed leaf and emerged in a beaker containing 20 ml of deionized water, at room temperature for 2 h. After this period, a reading of the electrical conductivity of the solution (EC1) was performed with the aid of a bench conductivity meter (TDS-3 digital meter). Then, the samples were subjected to heating in an autoclave at 121 °C for 20 min and, after cooling, a new final electrical conductivity reading (EC2) was performed. The electrolyte leakage index was determined considering the following formula: EC1/EC2 × 100.

#### Relative water content

Relative water content in the leaf (RWC) was determined by collecting three leaf discs (with approximately 129 mm^2^) of the first fully developed leaf, which were immediately weighed to measure tissue fresh matter (Fm). After that, the samples were rehydrated in deionized water for 6 h, to determine turgid matter (Tm), using paper towels to extract the excess water. Dry matter (Dm) was calculated after the discs had remained in a forced air circulation oven at 80 °C for 24 h. The relative water content values were determined by the equation proposed by Barrs and Weatherley^48^: [(Fm − Dm)/(Tm − Dm)] × 100.

#### Plant height and number of tillers

Plant height was measured considering the length from the base to the apex of the last leaf, and the number of tillers was counted.

#### Dry matter production

The plants were washed in running water, detergent solution (0.1% v:v), HCl solution (0.3% v:v) and deionized water. The plant material was dried in a forced air circulation oven (65 ± 5 °C) to constant mass and plant dry mass weight was determined.

#### Silicon analysis

The Si content in the shoot was measured by extracting the element according to the methodology described by Kraska and Breitenbeck^49^, and Si reading was performed by a spectrophotometer at 410 nm, as indicated by Korndörfer^41^. Si accumulation the shoots of the plants was calculated based on Si content and dry matter.

#### Carbon analysis

Total concentration of C in the shoots was determined by dry combustion (1000 °C), using an elemental analyzer (LECO Truspec CHNS) calibrated to the standard LECO 502–278 of wheat (C = 45.00% e N = 2.68%). Total N content was determined following the method of Bataglia et al.^50^.

Carbon efficiency was calculated using the equation: (dry matter)^2^/C accumulation in the plant^51^.

#### Statistical analysis

The collected data underwent analysis of variance by the F-test, and the averages were compared by Tukey’s test, both at 1 and 5% probability, using the SAS^®^ statistical software^52^.

## Results and discussion

### Biological damage from water deficit in forages

Reports on the tolerance to water deficit damage in the forage cultivars under study are scarce, especially in relation to N and C accumulation, Si effects, and physiological attributes.

**Figure 2:**
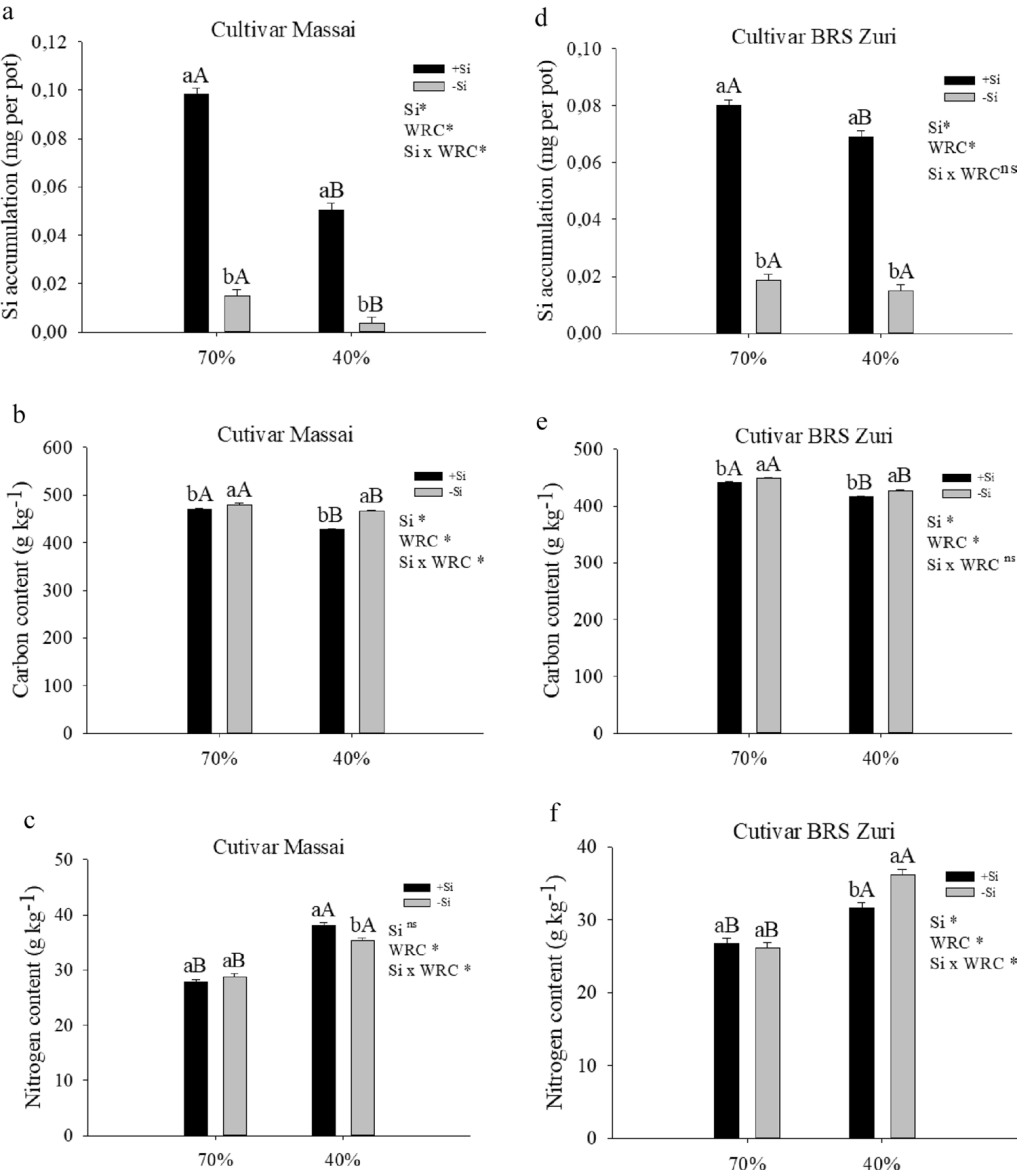
Silicon (Si) content (**a**, **d**), carbon (C) content (**b**, **e**) and nitrogen (N) content (**c**, **f**) in the aerial part of forage plants cultivated in soil with different soil water retention capacity (WRC) (70 and 40%) absence (− Si) and in the presence of silicon fertigation (+ Si). *Significant to 5% probability by the F test. Lowercase letters show differences in relation to Si and uppercase in relation to WRC. The bars represent the standard error of the mean, n = 6.

Pastures grown under water restriction with and without silicon showed a decreased cumulative amount of the beneficial element. However, pastures grown with or without water restriction that had received silicon had an increase in the cumulative amount of silicon (Fig. 2a,d). Carbon content decreased in pastures that had received silicon, regardless of water availability (Fig. 2b,e). Water restriction increased N content in both treatments with and without Si for both forages. Silicon fertigation only in plants with water restriction increased N content in cultivar Massai but decreased it in cultivar BRS Zuri (Fig. 2c,f).

The present study evidenced, especially with Si addition to the crop, that water deficit in the *P. maximum* pasture, regardless of cultivar, significantly impairs plant growth by changing homeostasis, i.e., decreasing the C:N ratio by reducing plant C content. This induces instability in the metabolism of the crop, especially in terms of physiological processes^31,53^. Thus, it was clear that water deficit aggravated physiological stress in the pastures due to an increase in electrolyte leakage, followed by a decrease in Fv/Fm. In other words, photosynthetic efficiency decreased in association with lower relative water content in the plant, which reduced the growth of both *P. maximum* cultivars*.*

**Figure 3:**
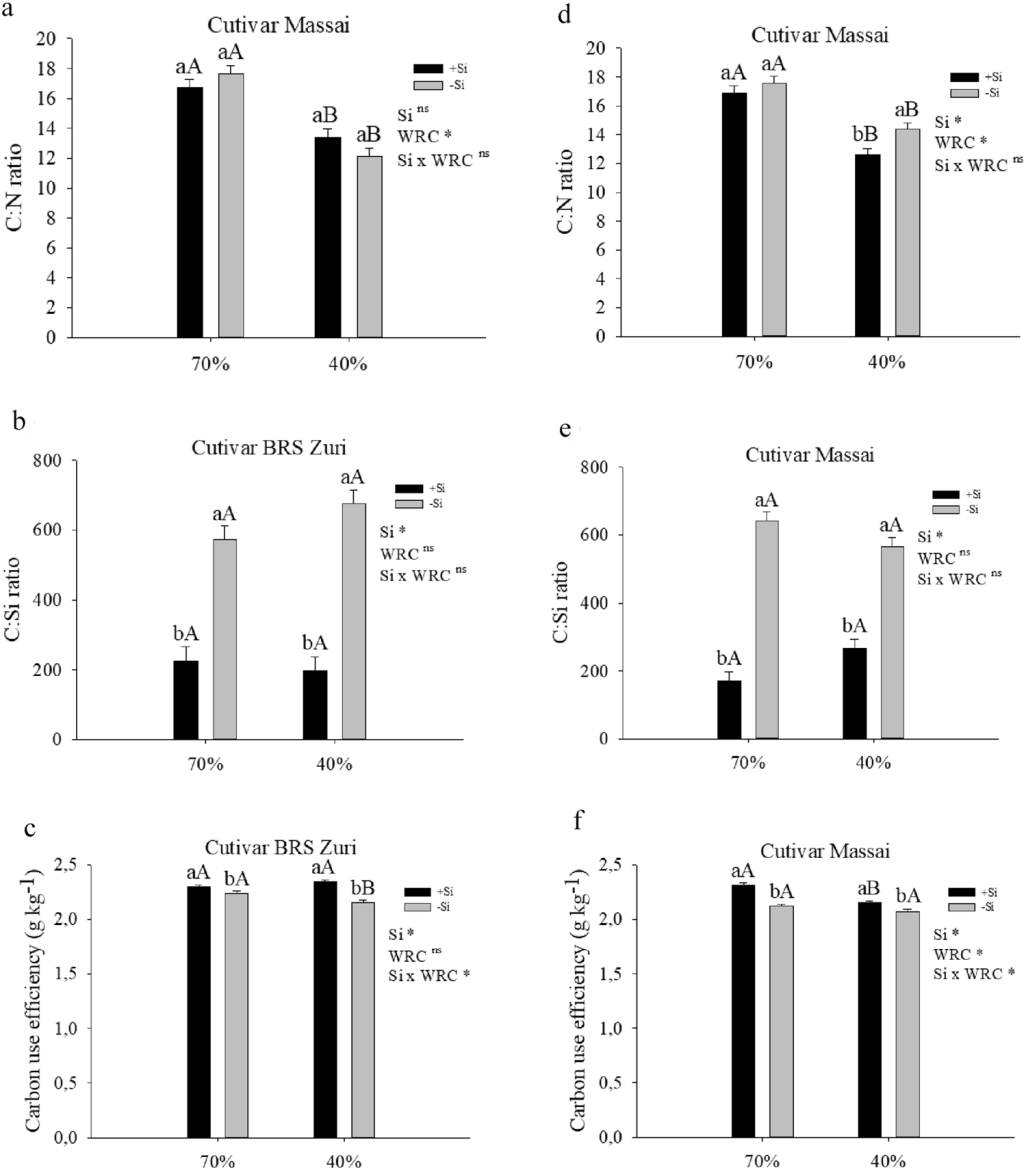
Ratio C:N (**a**, **d**), ratio C:Si (**b**, **e**) and carbon use efficiency (**c**, **f**) in the aerial part of forage plants cultivated in soil with different soil water retention capacities (WRC) (70 and 40%) %) absence (− Si) and in the presence of silicon fertigation (+ Si). *Significant at 5% probability. ns: not significant by the test F. Lowercase letters show differences in relation to Si and capitalization in relation to WRC. The bars represent the standard error of the mean, n = 6.

Water deficit in both pastures with and without silicon supply decreased the C:N ratio, except in cultivar Massai, in which the omission of silicon increased this ratio. In an adequate condition of water availability, there was no difference between the absence and presence of Si in the pastures (Fig. 3a,d). Other authors report the same results for different forages, such as sugarcane^53^. Water deficit in the pastures did not change the C:Si ratio, regardless of Si. In pastures with or without water deficit, silicon fertigation decreased the C:Si ratio (Fig. 3b,e).

Although this species has a high capacity for dry matter accumulation because it has a high protein content^54^, it is sensitive to drought^55^. Drought damage to plant growth, is due to the loss of stoichiometric stability of nutrients^56^, which balances the mass of various elements between plants and their environments^57^.

A promising alternative to mitigate water deficit damage in the pasture is the use of Si. This element plays a vital role in the physiological, metabolic, and/or functional processes of plants^58^ when properly absorbed by the crop. The present study evidences the high capacity of the pastures under study to absorb Si when under water restriction. This is because *P. maximum* is a Si-accumulating species (leaf Si content > 10 g kg^−1^), which means that these plants might have specific efficient carriers in the process of Si absorption (monosilicic acid)^37,59^.

### Biological benefits of silicon in mitigating water deficit in forage

The high Si absorption by the pastures was important because it was enough to change C and N contents in the pastures under water deficit, and consequently the C:N ratio. However, Si absorption varied depending on the cultivar. In cultivar Massai, the absorption of this element decreased due to an increase in N content, while the opposite occurred in cultivar BRS Zuri. This may have occurred because cultivar Massai has higher N absorption efficiency than BRS Zuri. One cultivar or species may have greater absorption efficiency than another because it has a more efficient nitrogen transporter. In other words, it has better kinetic indexes, such as low KM and minimum concentration, which is governed by genetics^31^.

The decrease in the C:Si ratio in plants grown under water restriction is a result of Si supply, which increased the absorption of this element and decreased C content in both pastures. Long et al.^28^ also reported the importance of silicon in elementary stoichiometry in a study with banana trees under water deficit.

The benefit of stoichiometric homeostasis reflected the high metabolic efficiency of C, that is, Si significantly increased C use efficiency in *P. maximum* pastures under water restriction (Fig. 3b,e). Other authors report this effect in *Brachiaria* spp. pastures under drought^25^ and in sugarcane plants without water stress^60^.

Carbon use efficiency (CUE) decreased in pastures with water restriction without silicon application. However, this variable increased in pastures where this element had been applied. In pastures under adequate water availability, silicon fertigation also increased CUE (Fig. 3c,f). Sugarcane plants under water deficit also showed decreased carbon use efficiency^53^. This increase in C use efficiency (Fig. 3c,f) by Si may have occurred in both pastures because there was a clear decrease in C content in plants grown under water restriction (Fig. 2b,e).

Hao et al.^29^ reported similar results in native grass species, in which high Si content correlated with low levels of C. This decrease in C content may have occurred because when absorbing the beneficial element, the plant applies an “exchange strategy” to C, particularly in cell wall components such as cellulose. This is because the energy cost of including Si in the carbon chain is lower than that of including C itself^61^. This strategy thus improves the homeostasis of resistance to water deficiency in pastures. Reports indicate that the increase in Si in plant tissues may decrease lignin synthesis in the cell wall, which has a high energy cost^62^; The plant uses a “low cost strategy” when occupying binding sites between cell wall components, providing similar structural resistance to that of lignin^63^.

**Figure 4:**
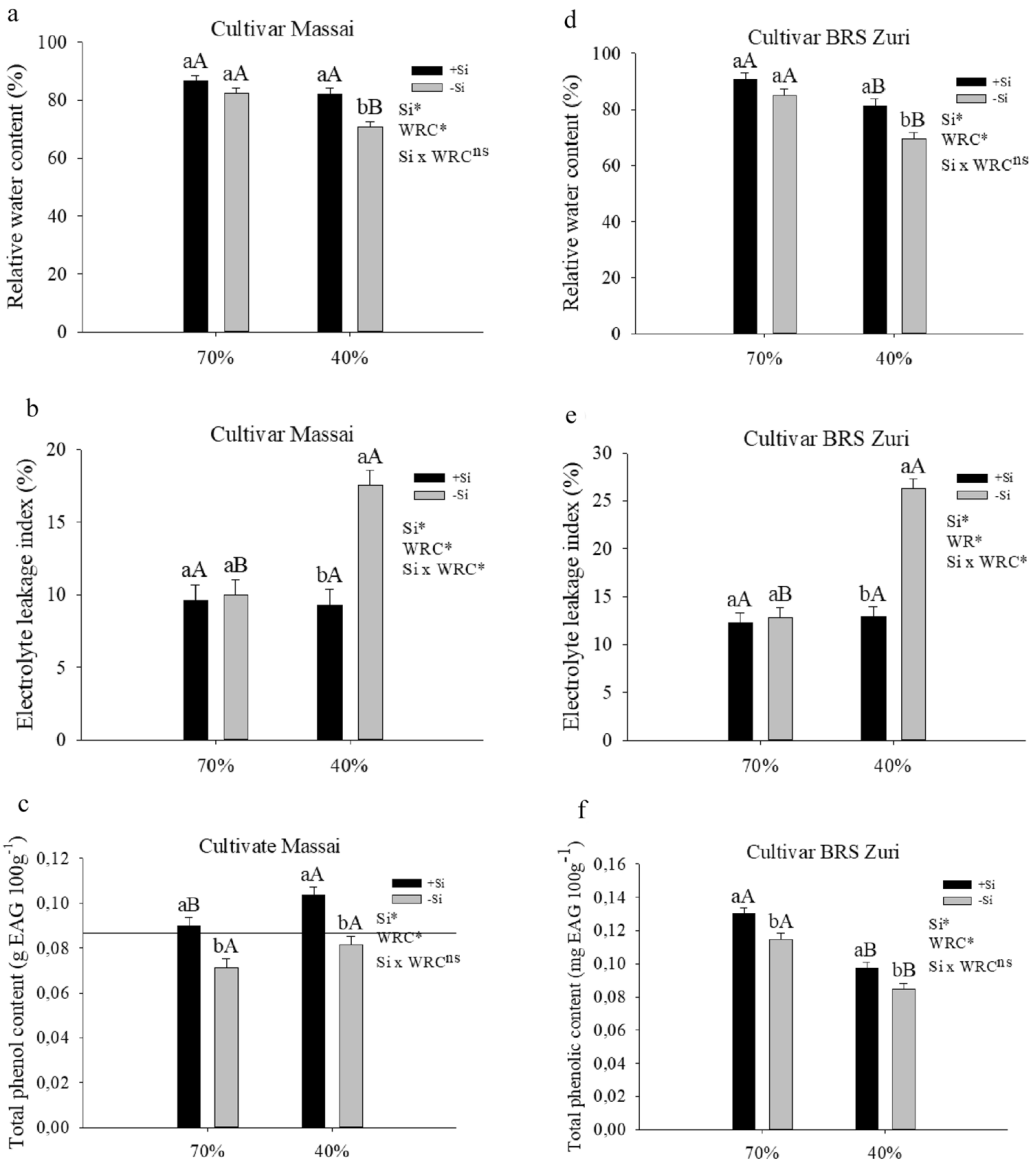
Relative water content (**a**, **d**), electrolyte leakage index (**b**, **e**) and Total phenolic content (**c**, **f**) of forage plants cultivated in soil with different soil water retention capacities (WRC) (70 and 40%) absence (− Si) and in the presence of silicon fertigation (+ Si). *Significant at 5% probability. ns: not significant by the test F. Lowercase letters show differences with respect to Si and uppercase in relation to WRC. The bars represent the standard error of the mean, n = 6.

These findings may support the promising role of Si in pasture management. This was evidenced from the effect of Si on elemental stoichiometry homeostasis in both forages grown under water restriction, which favored vital physiological processes by increasing the relative water content of the plant by approximately 14% (Fig. 4a,d). However, the effect of Si on the stoichiometric homeostasis of C might have induced energy savings in the plant, which is critical under water deficit conditions. Plants under water deficit have a limitation in the CO_2_ assimilation rate accompanied by an increase in the activity of another sink of absorbed energy, for example, photorespiration^30^. Studies on other crops confirm this finding, indicating a benefit of Si on stoichiometric homeostasis in plants under water deficit. Some examples are the studies of Rocha et al.^25^ on pasture, and Oliveira Filho et al.^26^ and Teixeira et al.^64^ on sugarcane.

Pastures under water deficit without silicon fertigation showed decreased relative water content in the plants. On the other hand, silicon fertigation increased the relative water content of forages under water deficit (Fig. 4a,d). Wang et al.^65^ performed a review to elucidate the effect of silicon on plant water transport processes. The authors indicated that silica deposition on leaf cuticle and stomata decreases water loss from transpiration under water deficit stress. However, accumulating evidence suggest that silicon maintains leaf water content not by reducing water loss, but rather through osmotic adjustments, enhancing water transport and uptake. According to the same authors, enhancement of stem water transport efficiency by silicon is due to silica depositing in the cell wall of vessel tubes, avoiding collapse and embolism.

The physiological improvement promoted by Si in attenuating water deficit in pastures probably correlates with the reduction of oxidative stress. In this sense, cell electrolyte leakage decreased (Fig. 4b,e), from the increase of the non-enzymatic antioxidant compound in both forages (Fig. 4c,f) or from the activity of antioxidant enzymes^66^. This reduces reactive oxygen species, which are common in plants under water deficit^67^.

Water deficiency affected the production of phenolic compounds depending on the cultivar. In Massai, this variable only increased with Si supply; in BRS Zuri, however, it decreased regardless of Si. Plants with silicon fertigation had increased phenolic compound content in pastures under both water availability conditions (Fig. 4c,f). Other authors have reported this effect of Si in increasing phenolic compounds in crops such as faba bean^68^ and sugar beet^69^. This supports the hypothesis that Si can attenuate the oxidative stress caused by water deficit by increasing the non-enzymatic antioxidant compound.

**Figure 5:**
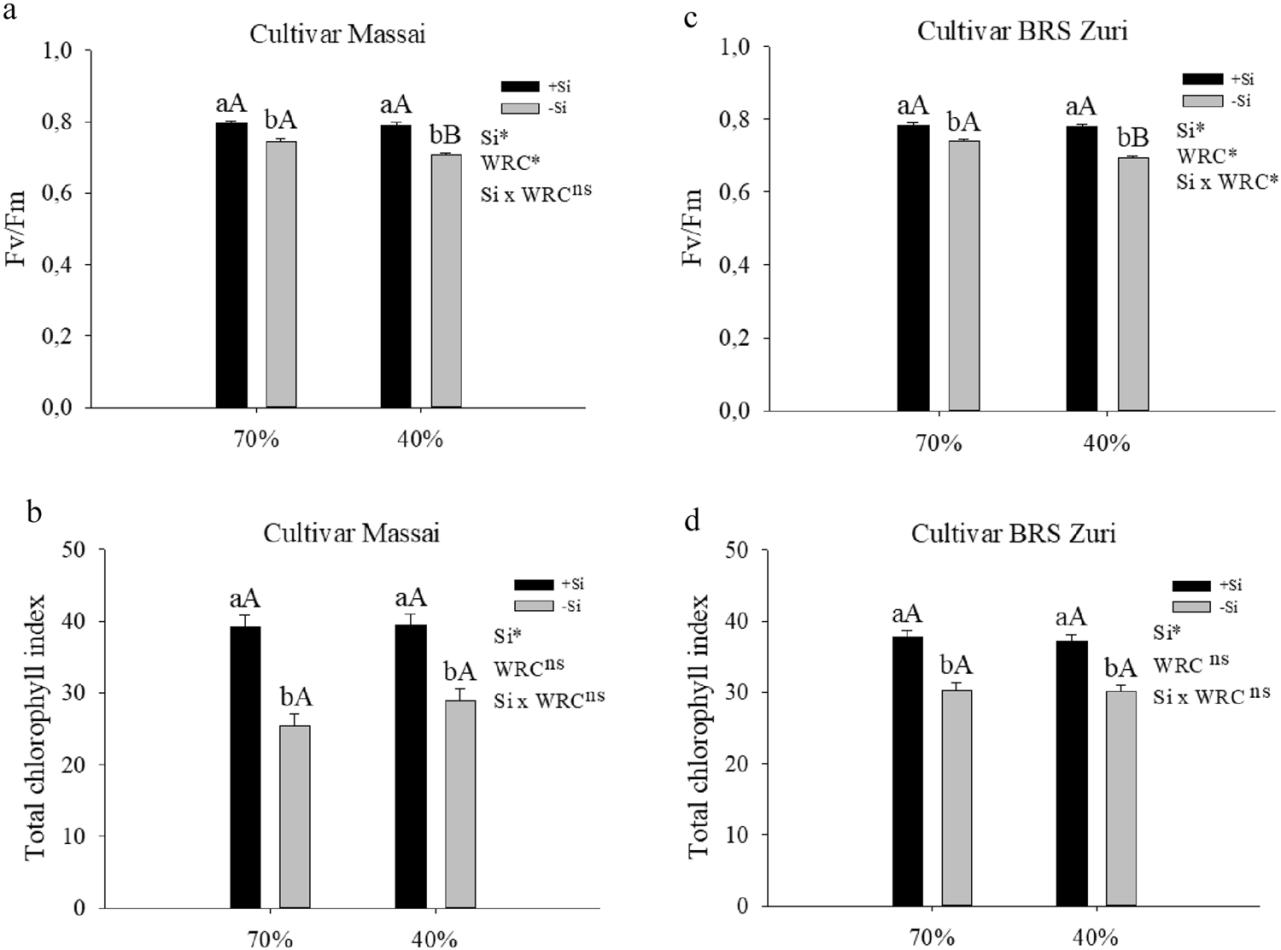
Quantum efficiency of photosystem II (Fv/Fm) (**a**, **c**) and total chlorophyll index (Chl a + b) (**b**, **d**) of forage plants grown in soil with different soil water retention capacities (WRC) (70 and 40%) absence (− Si) and in the presence of silicon fertigation (+ Si). *Significant at 5% probability. ns: not significant by the test F. Lowercase letters show differences in relation to Si and capitalization in relation to WRC. The bars represent the standard error of the mean, n = 6.

Exogenous application of Si protects the photosynthetic pigments from oxidative damage by reducing membrane lipid peroxidation. In peanut, this type of application either maintained or reduced H_2_O_2_^68^. Another effect of Si that demonstrates the attenuation of oxidative stress in pastures under water deficit was the increase in Fv/Fm; in other words, it favored photosynthetic efficiency. In both pastures, the condition of water restriction without silicon supply decreased the quantum efficiency of PSII (Fv/Fm). However, the supply of silicon in pastures, regardless of water condition, increased the photochemical efficiency of PSII (Fig. 5a,c).

The protection of photosynthetic pigments by Si is also indicative of decreased oxidative stress^58^. The present study evidenced this situation, as the beneficial element increased the total chlorophyll index in both forages under water deficit (Fig. 5b,d). Wang et al.^69^ reported that Si delays the degradation of chlorophyll–protein complexes, as the element alters the protein components of the thylakoid, thus optimizing the light collection and stability of PSI. Another benefit of Si would be an increase in osmoprotection as a result of the greater accumulation of metabolites, mainly sugars and sugar alcohols (talose, mannose, fructose, sucrose, cellobiose, trehalose, pinitol, and myo-inositol) and amino acids (glutamic acid, serine, histidine, threonine, tyrosine, valine, isoleucine, and leucine), as seen in peanut plants^68^.

### Si benefit on forage productivity under water deficit

**Figure 6:**
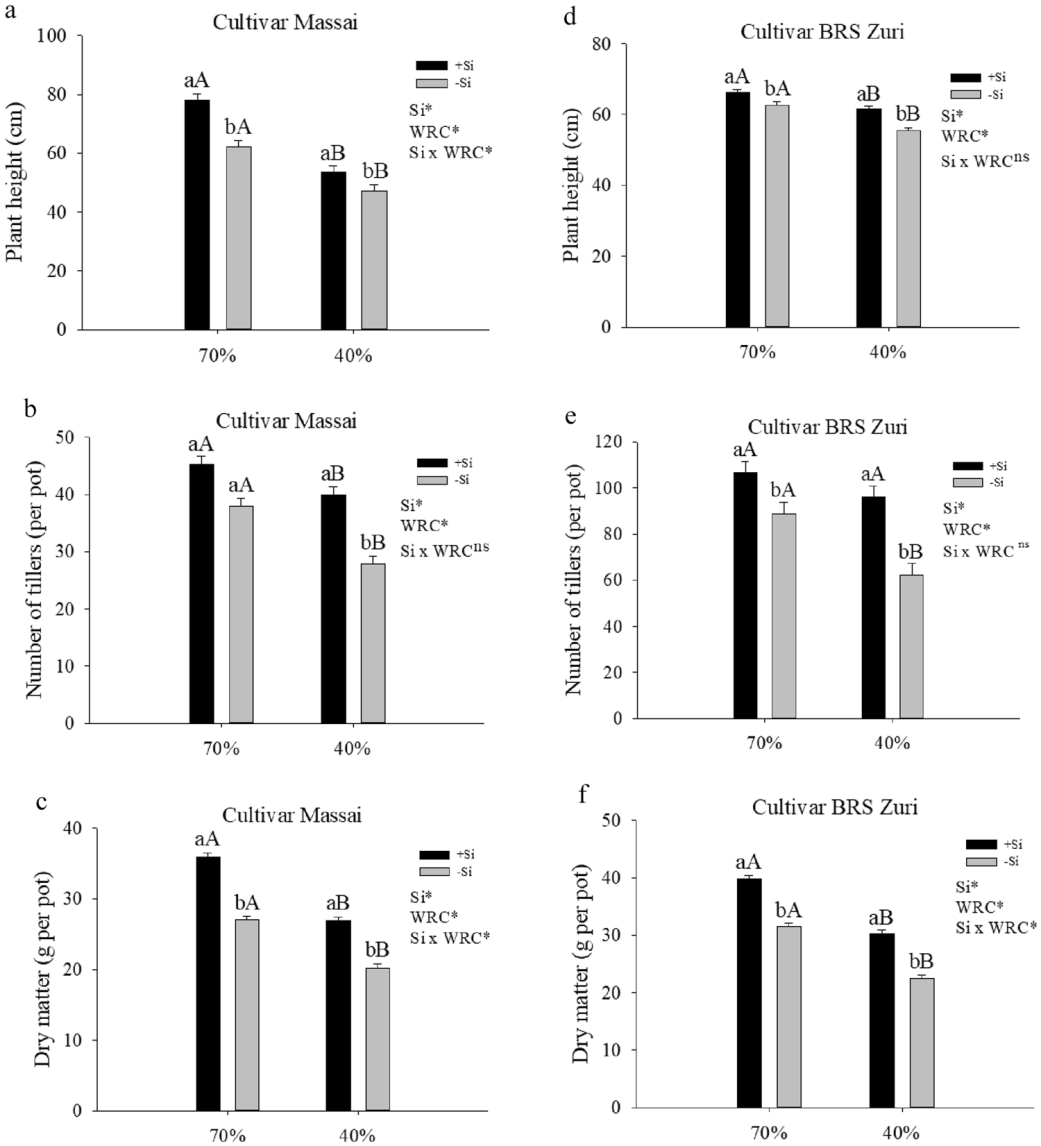
Plant height (**a**, **d**), number of tillers (**b**, **e**) and dry matter mass (**c**, **f**) of forage plants grown in soil with different soil water retention capacity (WRC) (70 and 40%) absence (− Si) and in the presence of silicon fertigation (+ Si). ns: not significant by the test F. Lowercase letters show differences in relation to Si and capitalization in relation to WRC. The bars represent the standard error of the mean, n = 6.

Water restriction with or without silicon supply decreased the height of both pastures, and silicon application in both water regimes increased plant height (Fig. 6a,d). Water restriction with or without silicon supply decreased the number of tillers in both pastures, except for the cultivar BRS Zuri that had received Si. Silicon application increased the number of tillers in both pastures in both water regimes, except for the cultivar Massai without water restriction (Fig. 6b,e). The dry weight of both pastures decreased under water deficit, regardless of silicon. However, the dry matter of the pastures increased after Si application, with or without water restriction (Fig. 6c,f).

**Figure 7:**
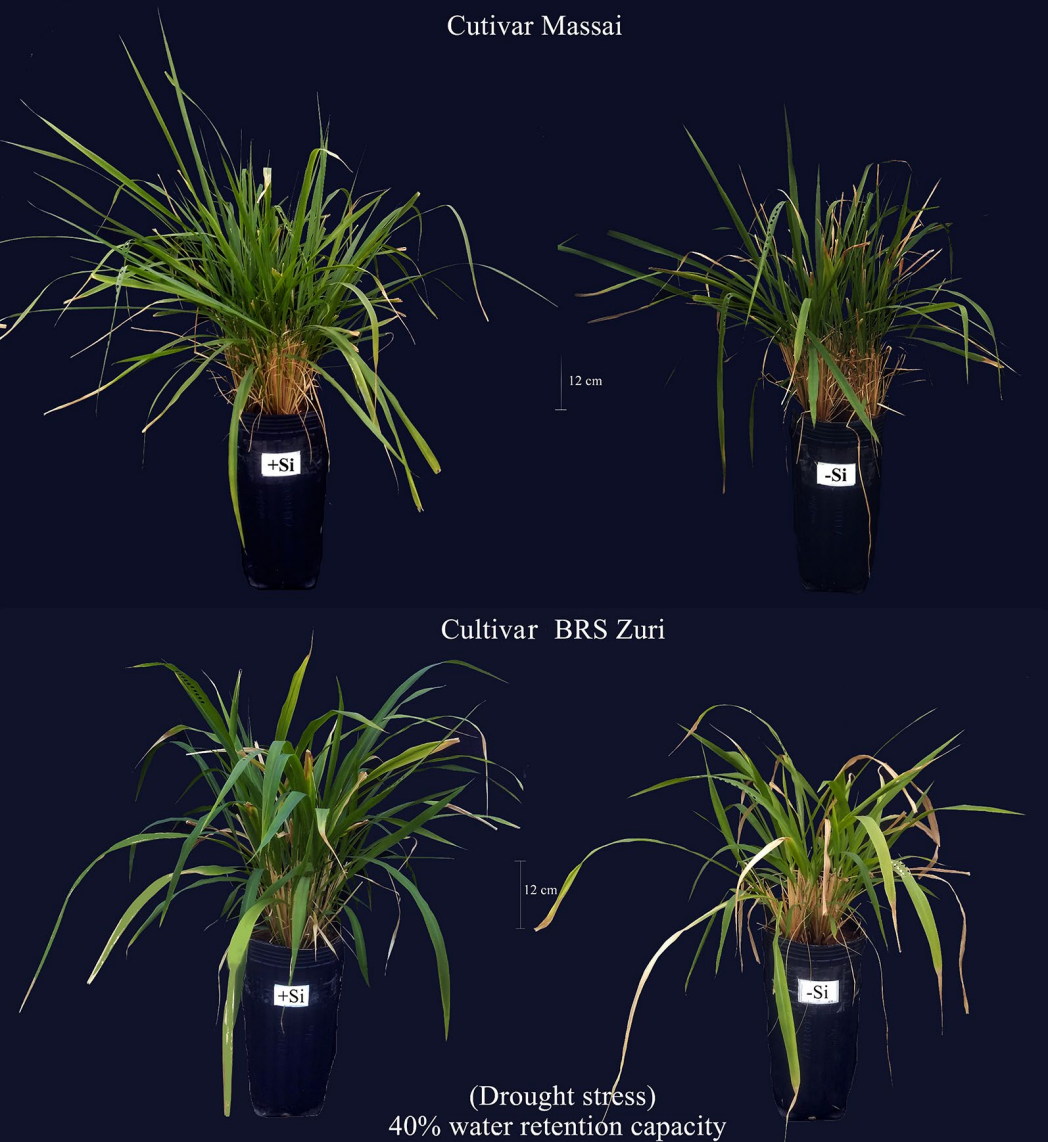
Figure of a forage plant in the condition of water deficit in the absence (− Si) and in the presence of silicon fertigation (+ Si) and a summary of its beneficial in the effects of the plant growth.

Thus, the mitigating effects of Si on the physiological processes of both pastures grown under water deficit were responsible for increasing forage growth by promoting an increase of 12% in plant height and 31% in the number of tillers, which is one of the main components of pasture production. This resulted in a 25% increase in dry matter accumulation in relation to the pasture without Si (Fig. 7). Other authors have also reported the mitigating effect of Si on water deficit with a view to increasing plant growth in forage crops^70^ and other crops like wheat^71^ and rice^72^.

The present study showed that the effect of Si on the attenuation of drought is not restricted only to physiological aspects involving increased plant water content and photosynthetic or biochemical efficiency. It also regulates elemental stoichiometric homeostasis as discussed above, confirming the biological strategy reported by Hao et al.^29^ in other forage grasses. Our study indicates that the line of research on the relationship between water deficit and Si in elementary stoichiometry is promising and should advance towards a better understanding of the multiple effects of this beneficial element on the plant.

**Figure 8:**
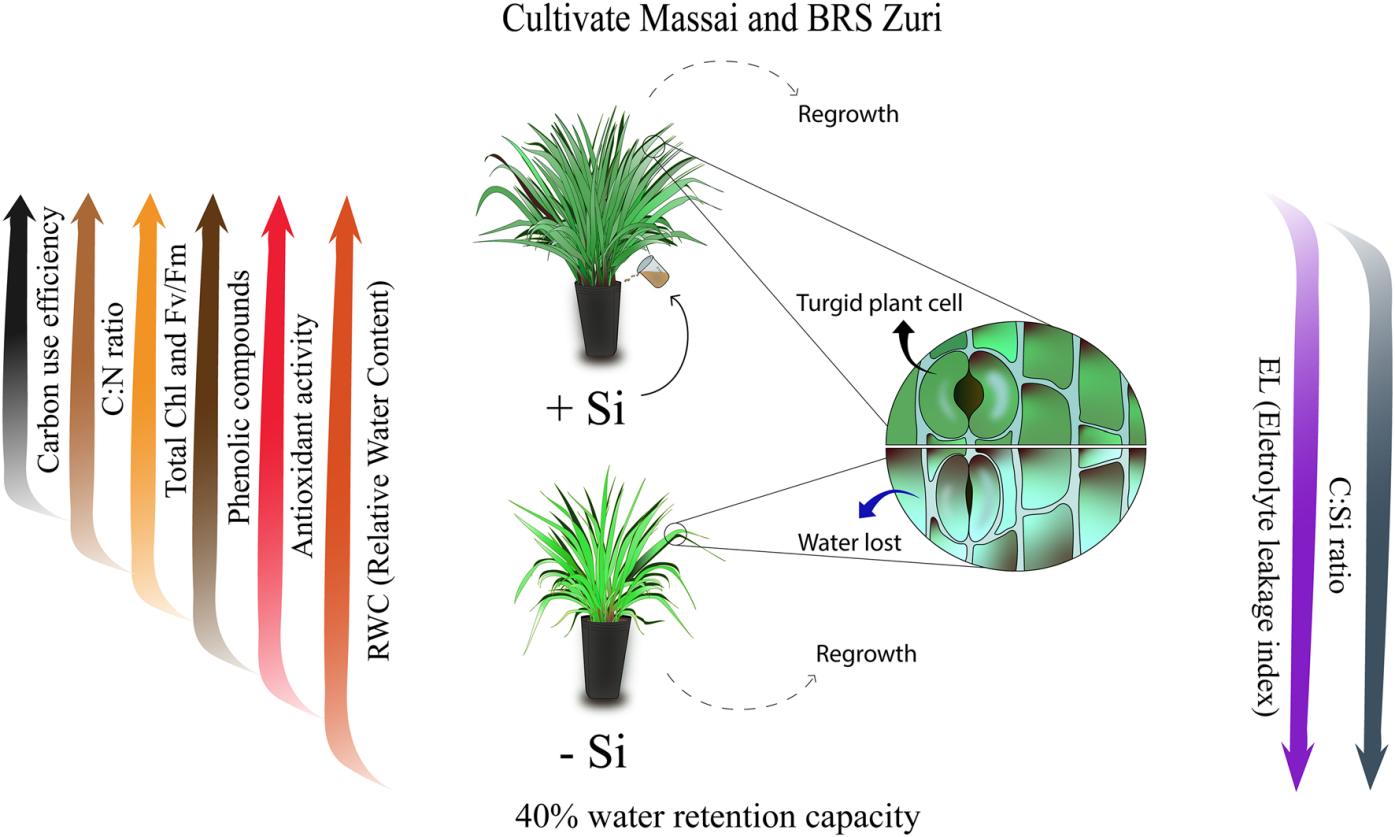
Benefits of Si in elementary stoichiometry and its relationship with physiological and biochemical aspects.

Animal production depends on the amount of biomass produced for grazing. The report of Habermann et al.^73^ has indicated that climate changes, such as droughts, are threatening pasture production and have a negative impact on animal and protein production. To solve this, the present research serves as a reference for Si fertigation management during the growth of *P. maximum*. This management consists of a sustainable alternative to improve production with greater nutritional balance even under soil water restriction, favoring water use efficiency in cultivation (Fig. 8). Moreover, Si has long-term potential to reduce the occurrence of droughts, favoring the sustainability of ecosystems. This is because the use of the beneficial element in the soil does not produce greenhouse gases, without negative impacts on the production environment^74,75^.

### Future perspectives

Peatlands and other terrestrial ecosystems represent large reservoirs and filters for Si, controlling Si transfer to the oceans. Land use change during the last 250 years has decreased soil Si availability by increasing export and decreasing Si storage due to higher erosion and a decrease in potentially Si-accumulating plants. Moreover, it has led to a twofold to threefold decrease of the base flow delivery of Si^76^. This raises concern over forage crops, reinforcing the need for silicate fertilization to explain the response of these species to the application of this element. Future perspectives would focuse on the benefits of Si in elementary stoichiometry and its relationship with physiological and biochemical aspects.

Studies should use, other forage species, especially dicotyledons sensitive to water deficit, which have different mechanisms for Si absorption. This will allow a better understanding of whether the Si mechanisms that attenuate drought in monocotyledons also occur in dicotyledons.

## Conclusion

*Panicum maximum* cultivars Massai and BRS Zuri are sensitive to water deficit without silicon supply, which causes disturbance in stoichiometric homeostasis and consequently in physiological aspects of the crop.

Water deficit was attenuated by Si, which had been applied via fertigation to favor the growth of the two cultivars of *P. maximum*. It stabilized the stoichiometric homeostasis of C:N and C:Si, which is responsible for increasing the conversion capacity of cumulative C in the plant into dry matter, thus contributing to some physiological processes in the plant—for example, increasing both the water content of plant tissues and photosynthetic efficiency.

The present study highlights the importance of plant nutrition associated with the physiological function of Si but neglected its effects on the stoichiometry of C and N, addressed in most research on pastures usually grown under water restriction.
